# Clinical importance of personality difficulties: diagnostically sub-threshold personality disorders

**DOI:** 10.1186/s12888-017-1200-y

**Published:** 2017-01-14

**Authors:** Max Karukivi, Tero Vahlberg, Kalle Horjamo, Minna Nevalainen, Jyrki Korkeila

**Affiliations:** 1 Department of Psychiatry, University of Turku and Turku University Hospital, Kiinamyllynkatu 4-8, FI-20520 Turku, Finland; 2 Unit of Adolescent Psychiatry, Satakunta Hospital District, Itäpuisto 11, FI-28100 Pori, Finland; 3 Department of Biostatistics, University of Turku, Lemminkäisenkatu 1, FI-20520 Turku, Finland; 4 Department of Public Health, University of Helsinki, PO Box 20, FI-00014 Helsinki, Finland; 5 Unit of Research and Development, Satakunta Hospital District, Sairaalantie 3, FI-28500 Pori, Finland; 6 Department of General Practice, University of Turku, Lemminkäisenkatu 1, FI-20520 Turku, Finland; 7 Psychiatric Care Division, Satakunta Hospital District, Sairaalantie 14, FI-29200 Harjavalta, Finland

**Keywords:** Personality assessment, Personality difficulty, Personality disorder, Quality of life

## Abstract

**Background:**

Current categorical classification of personality disorders has been criticized for overlooking the dimensional nature of personality and that it may miss some sub-threshold personality disturbances of clinical significance. We aimed to evaluate the clinical importance of these conditions. For this, we used a simple four-level dimensional categorization based on the severity of personality disturbance.

**Methods:**

The sample consisted of 352 patients admitted to mental health services. All underwent diagnostic assessments (SCID-I and SCID-II) and filled in questionnaires concerning their social situation and childhood adversities, and other validated tools, including the Beck Depression Inventory (BDI), Alcohol Use Disorders Identification Test (AUDIT), health-related quality of life (15D), and the five-item Mental Health Index (MHI-5). The patients were categorized into four groups according to the level of personality disturbance: 0 = No personality disturbance, 1 = Personality difficulty (one criterion less than threshold for one or more personality disorders), 2 = Simple personality disorder (one personality disorder), and 3 = Complex/Severe personality disorder (two or more personality disorders or any borderline and antisocial personality disorder).

**Results:**

The proportions of the groups were as follows: no personality disturbance 38.4% (*n* = 135), personality difficulty 14.5% (*n* = 51), simple personality disorder 19.9% (*n* = 70), and complex/severe personality disorder 24.4% (*n* = 86). Patients with no personality disturbance were significantly differentiated (p < 0.05) from the other groups regarding the BDI, 15D, and MHI-5 scores as well as the number of Axis I diagnoses. Patients with complex/severe personality disorders stood out as being worst off. Social dysfunction was related to the severity of the personality disturbance. Patients with a personality difficulty or a simple personality disorder had prominent symptoms and difficulties, but the differences between these groups were mostly non-significant.

**Conclusions:**

An elevated severity level of personality disturbance is associated with an increase in psychiatric morbidity and social dysfunction. Diagnostically sub-threshold personality difficulties are of clinical significance and the degree of impairment corresponds to actual personality disorders. Since these two groups did not significantly differ from each other, our findings also highlight the complexity related to the use of diagnostic thresholds for separate personality disorders.

## Background

Personality disorders are common chronic mental disorders that typically associate with problematic interpersonal relationships and a variety of social dysfunction [1]. According to epidemiological studies, the prevalence of a personality disorder diagnosis is 4 − 15% in general population, with a somewhat higher prevalence in males compared with females [2, 3]. Among psychiatric outpatients, the findings regarding prevalence vary, but a conservative estimate for western outpatient populations is approximately 40 − 50% [4].

One central instrument for diagnostic assessment of personality disorders is the Structured Clinical Interview for DSM-IV Personality Disorders (SCID-II) [5]. The SCID-II interview identifies the following personality disorders based on the DSM-IV-TR classification: borderline, antisocial, narcissistic, histrionic, avoidant, dependent, schizoid, schizotypal, paranoid, and obsessive-compulsive [6]. In general, the DSM-5 criteria for the personality disorders are essentially the same, only the text has been revised [7]. A core problem in the categorical approach is that personality characteristics are dimensional by nature [8]. The ten personality disorders identified in the classification have also been shown to include significantly overlapping characteristics [9]. Personality disorders can also be further categorized into three clusters, namely A (paranoid, schizoid and schizotypal), B (antisocial, borderline, histrionic, and narcissistic) and C (avoidant, dependent, and obsessive-compulsive).

Taking into account the dimensional nature of personality traits, the use of strict cutoff points is bound to miss those personality disturbances of clinical significance that fall slightly under the fixed diagnostic threshold. To improve the assessment of the clinical significance of personality disturbances, alternative methods for the interpretation of the SCID-II results have been developed. For example, Tyrer and Johnson (1996) developed a five-level cluster method that also takes into account those personality difficulties that fall a bit short of the threshold for an actual personality disorder diagnosis [10]. The categorization is as follows: 0 = No personality disturbance, 1 = Personality difficulty (one criterion less than threshold for personality disorder), 2 = Simple personality disorder (in one DSM cluster only), 3 = Complex personality disorder (two or more personality disorders in more than one DSM cluster), and 4 = Severe personality disorder (two or more personality disorders in more than one DSM cluster with one being antisocial personality disorder). It has been suggested that even a crude three-level dimensional approach (absent, sub-threshold traits, present) utilizing the results for SCID-II may be a more valid measure of psychosocial morbidity than the traditional categorical approach [11]. Recently, Kim et al. (2014) also used a modified four-level dimensional approach with a classification of personality disturbances ranging from “Personality difficulty” (the mildest) to “Severe personality disorder” (the most severe) [12].

Although the categorical approach is suggested to be favored by clinicians [13], a dimensional assessment offers certain benefits. First of all, personality disturbances below the diagnostic threshold may have a significant negative effect on the individual’s well-being and social functioning [14, 15]. Thus, in clinical settings, the identification of such disturbances may be crucial. Due to the nature of personality disorders, social dysfunction and comorbidity with Axis I disorders are also common [16]. The more complex and/or severe the personality disturbance, the stronger its negative effect on social dysfunction [15]. In all, the deteriorating effect of personality disorders on the quality of life is often significant and the burden related to them is comparable to severe somatic illnesses [17]. Personality disturbances often complicate the treatment of Axis I disorders [18] and appear to significantly increase the risk for relapses in Axis I disorders [19]. This effect is so prominent that, in his recent research, Tyrer (2015) hypothesized that personality dysfunction may be a core explanation for recurrent non-cognitive mental disorders [20].

The aim of the present study was to evaluate the clinical significance of diagnostically sub-threshold personality difficulties. For this, we tested a four-level dimensional categorization based on the severity of personality disturbance and simplified the Tyrer and Johnson’s criteria [10] by reducing the number of categories and omitting the DSM cluster criteria. We hypothesized that the diagnostically sub-threshold personality difficulties would be associated with a level of psychiatric morbidity and social dysfunction comparable to actual personality disorders. We also hypothesized that a higher level of personality disturbance would be associated with adverse childhood experiences, as well as higher psychiatric morbidity and social dysfunction.

## Methods

### Participants

**Fig. 1:**
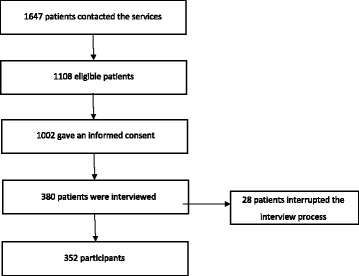
Flow chart of study participants

The analyzed sample was recruited from among 18 − 65-year-old patients attending psychiatric outpatient clinics within Satakunta Hospital District, Finland. The recruitment was conducted between years 2010 − 2014 and was limited to a) first-visit patients b) without any contact to mental health services during the preceding three years. In order to make the sample to bas representative as possible, the only two exclusion criteria were a) mental retardation and b) diagnosed neurologic illnesses. The flow chart for the participants is presented in Fig. 1. Diagnostic interviews were conducted after training for interviewing had taken place. The trained interviewers were psychiatric nurses and psychiatrists. The training took three days and included general information on psychopathology, theoretical basis for the Structured Clinical Interview for both DSM-IV Axis I (SCID-I) and Axis II (SCID-II) disorders and practical training. Thereafter, altogether 380 patients could be interviewed after the interviewer training was completed. The results for the SCID-II interviews were available for 352 patients and they formed the final sample. The participants differed from the non-participants only in terms of dwelling (p < 0.001), the participants being more often cohabiting (71.5% vs. 59.1%). Thus, the studied sample appeared to be representative for the whole sample. The participants were carefully assessed using interviews and self-assessment questionnaires.

### Measures

All participants were assessed with the SCID-I and SCID-II interviews [5, 21]. For the SCID-I, all diagnoses reaching the diagnostic threshold were recorded. Regarding the SCID-II, we used a simplified four-level modification of Tyrer and Johnson’s (1996) criteria for the classification of personality disturbance [10]. Our modifications were that instead of separate Complex and Severe categories, we used one Complex/Severe category, and we did not include the use of DSM clusters in our categories. The categorization was as follows: 0 = No personality disturbance, 1 = Personality difficulty (one criterion less than threshold for one or more personality disorders), 2 = Simple personality disorder (one personality disorder), and 3 = Complex/Severe personality disorder (two or more personality disorders or any borderline and antisocial personality disorder). Antisocial and borderline personality disorders were placed at the top of the hierarchy with the assumption that these personality disorders are determinants of other personality disorders.

Depressive symptoms were measured using the amended version of the Beck Depression Inventory (BDI-IA, here BDI) [22]. All 21 items are rated on a four-point Likert-type scale and the total score range is from 0 to 63. The psychometric properties of the scale have been shown to be good in several studies [23]. At least 80% of the items (18/21) had to be answered for the response to be included in the analyses. The missing values were estimated, using the mean value of the items answered, and the scores calculated in this way were rounded to the nearest integer.

Alcohol Use Disorders Identification Test (AUDIT) is a screening tool for hazardous drinking and alcohol dependence [24]. Several abbreviated versions have been developed on the basis of the original instrument and for the present study, we used the five-item version of the scale (AUDIT-PC) [25]. The total score range for the AUDIT-PC scale is 0 − 20 and a score of five or more suggests hazardous alcohol use. The instrument has shown good validity compared with the original scale [25].

Health-related quality of life (HRQOL) was measured using the 15D scale [26]. The 15D is a self-administered instrument that measures quality of life regarding a total of fifteen dimensions of health. The psychometrical properties of the instrument have been established as good in several studies [26]. The instrument can be used to provide a HRQOL profile or a single index number (range from 0 to 1) reflecting the overall HRQOL (0 = dead, 1 = full health). For the present study, we used the single index number as a measure for the HRQOL of the participants.

The five-item Mental Health Index (MHI-5) is derived from the widely-used 36-item Short-Form Health Survey Questionnaire (SF-36) and it is a general measure of emotional well-being [27]. The five statements are rated on a six-point Likert-type scale and scored between 1 and 6. The total raw score range is from 5 to 30, which is transformed, using linear transformation, into a variable ranging from 0 − 100. A higher score indicates better emotional well-being. The instrument has shown good validity and reliability [28].

Adverse childhood experiences were assessed with a 10-item questionnaire. The first six items were based on a questionnaire used in previous Finnish studies [29, 30] and the following four items: “Did a family member have a mental disorder?”, “Did someone in your family repeatedly use foul language, insult you or humiliate you while speaking to you?”, “Have you experienced repeatedly physical abuse in your childhood?”, and “Have you experienced sexual abuse in your childhood or adolescence?” Each of the five items had two response options (“yes” or “no”).

Since we aimed to assess the effect of personality disturbances on social dysfunction, several social variables based on self-reports were also included in the analyses. The variables were categorized as follows: basic education (comprehensive school dropout/comprehensive school/upper secondary school), studies (no vocational education/vocational education/polytechnic degree/academic degree), main occupation (working/rehabilitation or sick leave/child-care leave/other), dwelling (single/cohabitation/supported housing), financial situation (good or moderate/neither/quite poor or poor), number of children (none or one/two or more), number of chronic illnesses (none/one or more), and number of earlier treatment contacts (one/two/three or more). Regarding the variables for chronic illnesses and number of earlier treatment contacts, the questionnaire included a list of alternatives to choose from, including a blank space for additional information. The answers were then added up and categorized as presented above.

### Statistical analyses

The distributions of the study variables were assessed both statistically and graphically. The normally distributed variables are presented as means and standard deviations (SD) and the non-normally distributed variables are characterized using medians and interquartiles (IQR).

The group differences between the four personality disturbance levels were analyzed using one-way analysis of variance (ANOVA) for normally distributed variables, and the Kruskall-Wallis test was used for non-normally distributed variables. The differences in the distributions for both the categorized social variables and Axis I disorders across the four personality disturbance levels were compared using Chi-square tests.

In order to assess the differences between, in particular, the “Personality difficulty” and “Simple personality disorder” levels, a series of post hoc analyses was conducted. In these analyses, Chi-square tests were used for categorized variables. Furthermore, Tukey’s method was used for normally distributed variables and the Dwass, Steel, Critchlow-Flinger method (DSCF) for non-normally distributed variables.

The internal consistency reliability for the BDI, AUDIT, and MHI-5 scales was calculated using the Cronbach’s alpha. The scores showed good internal consistency: 0.90 for BDI, 0.78 for AUDIT, and 0.90 for MHI-5. In all analyses, p-values <0.05 were considered statistically significant. Statistical analyses were carried out using SAS System for Windows, version 9.4 (SAS Institute Inc., Cary, NC, USA).

## Results

The proportion of patients reaching the diagnostic threshold for at least one personality disorder was 47.2% (*n* = 166), and altogether 24.4% (*n* = 86) of patients were diagnosed with two or more personality disorders. The distributions of the categorized variables according to the level of personality disturbance are presented in Table 1. Several statistically significant differences were observed and the presence of a “Complex/Severe personality disorder” was particularly associated with lower education and a more difficult financial situation. The descriptive statistics for the continuous variables are presented in Table 2.

**Table 1:** Categorized variables and their distributions according to the level of personality disturbance

				No personality disturbance		Personality difficulty		Simple personality disorder		Complex/severe personality disorder		
Variable		n	%	n	%	n	%	n	%	n	%	P^a^
Gender (*n* = 352)	Female	118	33.5	37	27.4	18	35.3	24	30.0	39	45.4	0.042
	Male	234	66.5	98	72.6	33	64.7	56	70.0	47	54.6	
Basic education (*n* = 354)	Comprehensive school dropout	26	7.1	15	11.2	1	2.0	4	5.0	6	6.9	0.007
	Comprehensive school	227	62.4	67	50.0	36	70.6	49	61.3	64	73.6	
	Upper secondary school	111	30.5	52	38.3	14	27.5	27	33.8	17	19.5	
Studies (*n* = 335)	No vocational education	84	24.4	18	14.0	9	18.4	23	30.3	30	37.0	0.001
	Vocational education	143	41.5	53	41.1	27	55.1	31	40.8	32	39.5	
	Polytechnic degree	92	26.7	44	34.1	9	18.4	20	26.3	13	16.1	
	Academic degree	26	7.5	14	10.9	4	8.2	2	2.6	6	7.4	
Main occupation (*n* = 364)	Working	92	25.3	43	31.6	14	27.5	14	17.7	19	22.1	0.030
	Rehabilitation/Sick leave	162	44.5	53	39.0	26	51.0	34	43.0	44	51.2	
	Child-care leave	21	5.8	11	8.1	4	7.8	2	2.5	3	3.5	
	Other	89	24.5	29	21.3	7	13.7	29	36.7	20	23.3	
Dwelling (*n* = 358)	Single	103	28.0	36	26.3	13	25.5	18	22.2	33	37.5	0.19
	Cohabitation	263	71.5	99	72.3	38	74.5	63	77.8	55	62.5	
	Supported housing	2	0.5	2	1.5	0	0.0	0	0.0	0	0.0	
Financial situation (*n* = 354)	Good/Moderate	101	28.4	54	40.9	19	37.3	12	15.8	16	18.8	<0.001
	Neither	108	30.4	45	34.1	10	19.6	28	36.8	25	29.4	
	Quite poor/Poor	135	38.1	33	25.0	22	43.1	36	47.4	44	51.8	
Number of children (*n* = 360)	None or one	192	51.9	71	51.1	27	52.9	45	55.6	49	55.7	0.89
	Two or more	178	48.1	68	48.9	24	47.1	36	44.4	39	44.3	
Number of chronic illnesses (*n* = 360)	None	121	32.7	49	35.3	12	23.5	26	32.1	25	28.4	0.42
	One or more	249	67.3	90	64.8	39	76.5	55	67.9	63	71.6	
Number of earlier treatment contacts (*n* = 289)	One	152	52.6	60	58.3	25	56.8	40	59.7	27	36.0	0.047
	Two	86	29.8	30	29.1	12	27.3	15	22.4	29	38.7	
	Three or more	51	17.6	13	12.6	7	15.9	12	17.9	19	25.3	

^a^Chi-square test

**Table 2:** The associations of the level of personality disturbance with the continuous variables

	No personality disturbance (I)		Personality difficulty (II)		Simple personality disorder (III)		Complex/severe personality disorder (IV)			Post hoc test^c^
Variable	N	Mean (SD) or Median [IQR]	N	Mean (SD) or Median [IQR]	N	Mean (SD) or Median [IQR]	N	Mean (SD) or Median [IQR]	P	
Age	135	40.95 (11.79)	51	40.18 (10.80)	80	38.60 (12.59)	86	40.24 (12.44)	0.59^a^	None
MHI-5	133	57.89 (21.91)	49	41.88 (21.53)	71	44.17 (19.81)	77	37.82 (19.73)	<0.001^a^	I vs. II, I vs. III, I vs. IV
15D	129	0.81 (0.10)	49	0.76 (0.10)	78	0.76 (0.11)	80	0.72 (0.12)	<0.001^a^	I vs. II, I vs. III, I vs. IV, III vs. IV
BDI	135	16.72 (8.85)	51	22.38 (8.28)	80	22.56 (9.06)	85	26.06 (9.23)	<0.001^a^	I vs. II, I vs. III, I vs. IV, III vs. IV
AUDIT	135	3.00 [5.00]	51	4.00 [7.00]	80	3.00 [5.50]	86	5.00 [7.00]	0.010^b^	I vs. IV
Number of Axis I diagnoses	135	1.00 [1.00]	51	2.00 [2.00]	80	2.00 [2.00]	86	3.00 [1.00]	<0.001^b^	I vs. II, I vs. III, I vs. IV, III vs. IV

*SD* Standard deviation, *IQR* Interquartile range, *MHI-5* Five-item Mental Health Index, *BDI* Beck Depression Inventory, *AUDIT* Alcohol Use Disorders Identification Test

^a^One-way ANOVA

^b^Kruskall-Wallis test

^c^Significant differences (p < 0.05) in pair-wise post hoc tests. Tukey’s method for Age, MHI-5, 15D, and BDI; Dwass, Steel, Critchlow-Flinger method for AUDIT and Number of Axis I disorders

On the basis of these results, we conducted a series of post hoc analyses in order to assess, in particular, to what extent the “Personality difficulty” and “Simple personality disorder” levels differed from each other. Regarding the continuous variables, no statistically significant differences between these two groups were observed (Table 2). When the two groups were compared with the “Complex/Severe personality disorder” group, a significant difference was found only for the number of Axis I disorders. In all, an increase in the severity of the personality disturbance was associated with an increase in the BDI and AUDIT scores and, correspondingly, a decrease in the MHI-5 and 15D scores. Additionally, the number of Axis I diagnoses increased concurrently.

The differences in the distribution of Axis I disorders according to the level of personality disturbance are presented in Table 3. Significant differences were observed in the distributions for the depressive, substance use, and anxiety disorders with the likelihood for an Axis I disorder increasing along with the severity of the personality disturbance. However, the differences between the “Personality difficulty” and “Simple personality disorder” levels were not significant.

**Table 3:** The associations of the level of personality disturbance with the diagnosed Axis I disorders

		No personality disturbance		Personality difficulty		Simple personality disorder		Complex/severe personality disorder		
Diagnosis		N	%	N	%	N	%	N	%	P^a^
Bipolar disorder	Yes No	13 127	9.3 90.7	8 43	15.7 84.3	14 67	17.3 82.7	16 72	18.2 81.8	0.17
Depressive disorders	Yes No	91 49	65.0 35.0	42 9	82.3 17.7	60 21	74.1 25.9	72 16	81.8 18.2	0.017
Other mood disorders	Yes No	1 139	0.7 99.3	0 51	0.0 100.0	4 77	4.9 95.1	1 87	1.1 98.9	0.12
Psychotic disorders	Yes No	5 135	3.6 96.4	0 51	0.0 100.0	2 79	2.5 97.5	1 87	1.1 98.9	0.53
Substance use disorders	Yes No	29 111	20.7 79.3	19 32	37.2 62.8	24 57	29.6 70.4	41 47	46.6 53.4	<0.001
Anxiety disorders	Yes No	47 93	33.6 66.4	23 28	45.1 54.9	39 42	48.1 51.9	51 37	58.0 42.0	0.003
Somatoformic disorders	Yes No	3 137	2.1 97.9	2 49	3.9 96.1	3 78	3.7 96.3	8 80	9.1 90.9	0.11
Eating disorders	Yes No	6 134	4.3 95.7	3 48	5.9 94.1	5 76	6.2 93.8	5 83	5.7 94.3	0.89
Adjustment disorders	Yes No	3 137	2.1 97.9	0 51	0.0 100.0	0 81	0.0 100.0	1 87	1.1 98.9	0.58

^a^Chi-square test

The number of adverse childhood events was significantly associated with the severity of the personality disturbance (χ2 = 28.08, df = 9, p < 0.001) (Table 4). When assessed separately, emotional neglect (χ2 = 14.26, df = 3, p < 0.05), physical neglect (χ2 = 11.07, df = 3, p < 0.05) and sexual abuse (χ2 = 11.07, df = 3, p < 0.05) were all significantly associated with the severity of the personality disturbance.

**Table 4:** The associations of the level of personality disturbance with childhood adversities

		No personality disorder		Personality difficulty		Simple personality disorder		Complex personality disorder		P^a^
Number of traumatic events in childhood		n	%	n	%	n	%	n	%	
0		31	24.2	10	21.2	10	14.7	7	15.7	<0.001
1		23	18.0	8	17.0	8	11.8	8	10.5	
2-3		35	27.3	14	29.8	14	20.6	15	19.7	
≥4		39	30.5	15	31.9	15	22.1	46	60.5	
		128	100.0	47	100.0	68	100.0	76	100.0	
Emotional neglect^b^	Yes	23	7.6	7	2.3	12	4.0	29	9.6	0.003
	No	279	92.4	295	97.7	290	96.0	273	90.4	
Physical abuse^b^	Yes	16	5.2	4	1.3	9	2.9	21	6.8	0.011
	No	291	94.8	303	98.7	298	97.1	286	93.2	
Sexual abuse^b^	Yes	7	2.2	3	1.0	8	2.6	15	4.8	0.009
	No	305	97.8	209	99.0	304	97.4	297	95.2	

^a^Chi-square test

^b^Due to missing data: Emotional neglect *n* = 302, Physical abuse *n* = 307, and Sexual abuse *n* = 312

Significant differences in the categorical variable distributions were found between the “Personality difficulty” and “Simple personality disorder” levels only for main occupation (*p* = 0.022) and financial situation (*p* = 0.013). In the “Simple personality disorder” group, the main occupation was more often “other” and the financial situation of the patients was worse. The differences for both of these groups were minimal when compared with the “Complex/Severe personality disorder” group. No significant differences between the “Personality difficulty” and “Complex/Severe personality disorder” groups were observed. Between the “Simple personality disorder” and “Complex/Severe personality disorder” groups, significant differences were only found for dwelling (*p* = 0.030) and the number of earlier treatment contacts (*p* = 0.018). Belonging to the latter group was associated with living alone and a higher number of treatment contacts.

## Discussion

The main finding of the present study was that diagnostically sub-threshold personality difficulties are significantly associated with marked psychiatric symptoms and negative effects on subjective well-being. The fact that our four-level dimensional approach was not capable of differentiating, in particular, between the “Personality difficulty” and “Simple personality disorder” groups, emphasizes precisely the core problem associated to the diagnostic thresholds for the separate personality disorder categories. Our results indicate that personality difficulties falling a bit under of the diagnostic thresholds are of clinical significance and the associated impairment equals to actually diagnosed single personality disorders. Additionally, our results indicate that an increase in the severity of personality disturbance is associated with an increase in psychiatric morbidity and social dysfunction. For psychiatric morbidity, HRQOL, and social dysfunction, it appears that the overall level of personality disturbance is of greater importance than separate personality disorder diagnoses.

The association between the severity of a personality disorder and psychiatric symptoms has been repeatedly reported (e.g., [31]). Yang et al. (2010) conducted an extensive analysis in a large (*n* = 8391) general population sample, and found the level of personality pathology to be significantly associated with a wide variety of variables representing psychiatric morbidity and social dysfunction [15]. However, they used only SCID-II screening questionnaires, while the results in the present study were based on full SCID interviews. Kim et al. (2014) studied a sample of psychiatric outpatients and inpatients using a four-level dimensional approach [12]. They observed statistically significant differences for trait anxiety, depressive symptoms and social dysfunction between the groups and those with a more severe personality disturbance were clearly worse off. In the present study, we found statistically significant differences for both the BDI and AUDIT scores across the personality disturbance levels, with the amount of symptoms increasing along with the level of personality disturbance. Additionally, the number of Axis I disorders increased in tandem. However, the differences between the personality disturbance levels were minimal, in particular, between the “Personality difficulty” and “Simple personality disorder” levels. As to co-morbidity, it was found that anxiety disorders and substance use disorders were particularly emphasized for the “Complex/Severe personality disorder” level.

In terms of childhood adversities and the social functioning variables, patients with a “Complex/Severe personality disorder” were almost invariably worst off. When compared with the “No personality disturbance” level, patients with “Personality difficulty” or a “Simple personality disorder” showed a higher degree of social dysfunction, but the differences between these two dimensions were marginal. The MHI-5 scores indicated that the patients’ psychiatric illnesses had a major impact on their emotional well-being regardless of the level of personality disturbance. The MHI-5 scores were well below the recommended cut-offs for the identification of common mental disorders [32].

For the HRQOL, in a study by Pirkola et al. (2009), the 15D scores were 0.91 − 92 for general population, but for different current mental illnesses they were as follows: depressive disorder 0.80, anxiety disorder 0.80, and alcohol dependence 0.89 [33]. A comorbid depressive and anxiety disorder was associated with the lowest 15D score: 0.78. In another population study by Saarni et al. (2010), the 15D score was between 0.82 − 0.87 for psychotic disorder patients [34]. Compared to these findings, the patients in the present study were worse off regarding their HRQOL. The severity of the personality disturbance had a significant negative effect on the HRQOL and the patients with complex personality disorders had very low 15D scores (0.72). The differences between the “Personality difficulty” and “Simple personality disorder” dimensions were scanty. In all, our results further strengthen the association between the severity of personality disturbance and psychiatric morbidity and overall well-being.

Our results are an addition to the increasing amount of literature emphasizing the clinical importance of, not only diagnosed personality disorders, but also milder personality disturbances. Yang et al. (2010) found diagnostic sub-threshold personality difficulties to be significantly associated with mental disorder symptoms and social dysfunction [15]. However, according to the current diagnostic criteria, these difficulties would not allow the use of a diagnosis. We found that the “Personality difficulty” level was associated with significant psychiatric symptoms and social difficulties. The symptoms and impairment were roughly at the same level as for the “Simple personality disorder” level, partly even worse. However, particularly when assessing milder personality difficulties, it is important to observe that present Axis I disorders also might emphasize certain personality difficulties [35]. The participants in our study were outpatients who were newly admitted to mental health services. Given the significant effect of personality difficulties on the prognosis of Axis I disorders [14, 19, 20], paying attention to these difficulties right from the beginning may have a major impact on the subsequent course of the treatment.

It has been proposed that the upcoming ICD-11 diagnostic criteria would utilize a more dimensional approach to personality disorders and thus, enable clinicians to take better into account the dimensional nature of personality and reduce the problem associated with the overlap of the different disorder categories [8, 9, 20, 36] The currently suggested personality disorder levels are: mild personality disorder, moderate personality disorder, and severe personality disorder [1]. “Mild personality disorder” is suggested to be used as an additional entity to denote characteristics that significantly influence a person’s health status and the use of health services, which are in general classified as Z codes under the current ICD-10 [37]. Although the criteria also include the assessment of five trait domains, such as negative affective features, the primary dimensional classification is the relevant one from the clinical point of view. In a study by Morgan et al. (2013), the authors observed that dimensional approaches indeed appeared to be of most significance particularly in milder personal disturbances [38]. However, it may be that we have to rethink the whole concept of personality disorders as Axis II disorders. Using a factor analysis, Kotov et al. (2011) suggested that, in terms of co-morbidity patterns, most of the personality disorders should be placed in Axis I [39].

Dimensional assessment of personality disturbances has additional benefits. Because of their effect on interpersonal relationships, personality disturbances often complicate doctor-patient relationships and may thus significantly hinder the patients’ possibility to get the help they need (e.g., [40]). The identification of these disturbances and elaborating on their manifestation and multiple links, may open up new treatment possibilities and decrease the mutual frustration. Personality disorders are often complicated, not only by Axis I disorders, but also by other personality disturbances and disorders. In the present study, one fourth of patients were diagnosed with two or more personality disorders. In these cases, treatment methods that are aimed only at one separate personality disorder are seldom worthwhile. However, the majority of intervention literature are based on these kind of samples. The dimensional approach to personality disorders may help to develop treatments that take the overall level of disturbance better into account. Personality difficulties may generally act more as a hindering factor for treatment, while complex/severe personality disorders clearly require more tailored and comprehensive treatment strategies.

Compared with the present classification of personality disorders, a simple dimensional categorization may also be easier for patients and their families to approach and adopt, and furthermore, improve adherence to treatment. One additional benefit of a dimensional assessment is the reduced stigma associated with personality disorders [15]. Indeed, the reduction of stigma is justified. Personality disorders are typically perceived as chronic and treatment-resistant, but recent longitudinal studies indicate that they remit more often and faster than assumed [41].

The present study has some limitations. The setting is cross-sectional and therefore precludes the possibility of drawing conclusions about causality. The studied sample only included a part of all first-visit patients eligible to the study, but nevertheless it appeared to be representative. Although we had a firm basis for the “Personality difficulty” criteria, they required a rather high level of personality disturbance and thus, resulted in a group with a rather high level of psychiatric morbidity and social dysfunction. When compared with the study by Kim et al. (2014), a difference can be observed, for example, in the BDI scores for the “Personality difficulty” group, which were markedly higher in the present study (14.06 vs. 22.98) [12]. Thus, it can be argued that our “Personality difficulty” patients rather represented patients with mild personality disorders. Additionally, since our categorization did not differentiate “Personality difficulty” and “Simple personality disorder” patient groups, the clinical feasibility of these criteria is limited. The categorization of all antisocial and borderline personality disorders to the “Complex/Severe personality disorder” group can also be questioned. However, only 17.8% (8/45) of the patients with borderline personality disorder and 36.4% (4/11) with antisocial disorder had a simple personality disorder. Additionally, as noted above, compared with others, these two disorders characteristically are complex personality disorders.

## Conclusions

In conclusion, our results lend support to the dimensional nature of personality disturbances and the association of the severity of the disturbance with higher psychiatric morbidity and social dysfunction. Our results also highlight the complexity related to the use of diagnostic thresholds for separate personality disorders. If the patients that meet the diagnostic threshold for a personality disorder do not significantly differ from the patients who do not meet the threshold criteria, the value of such thresholds has to be questioned. Although certain personality disorder categories, as well as the personality trait domains in the proposed classification, will surely provide tools for fruitful academic research also in the future, from a clinical point of view, a dimensional approach that takes into account a broader view of the personality disturbance may be feasible.
